# Social environment, low-carbon cognition and low-carbon consumption behaviors of youth groups: evidence from Xizang, China

**DOI:** 10.3389/fpsyg.2025.1494761

**Published:** 2025-02-12

**Authors:** Huifang Ma, Qin Chen

**Affiliations:** ^1^Politics and Public Administration College, Qinghai Nationalities University, Xining, China; ^2^Guangdong Key Laboratory of Mental Health and Cognitive Science, South China Normal University, Guangzhou, China; ^3^School of Public Policy and Management, Tsinghua University, Beijing, China

**Keywords:** the youth group, low-carbon consumption, influence factor, low-carbon cognition, social environment

## Abstract

**Introduction:**

Global warming has profoundly transformed the natural environment and significantly impacted people’s production methods, thereby promoting low-carbon consumption behaviors. While numerous scholars have examined the factors influencing low-carbon consumption behavior, their analyses predominantly rely on classical theoretical frameworks such as planned behavior theory, value-belief-norm theory and The ABC attitude theory. However, there is a notable scarcity of studies investigating the relationship between the social environment and low-carbon behaviors among youth groups. To address this gap in the literature, we aim to explore how the social environment influences youth groups’ low-carbon consumption behaviors, as well as identify the mechanisms through which this influence may manifest.

**Methods:**

This study examines the factors influencing low-carbon consumption behavior from three perspectives: the social environment, low-carbon cognition, and conformity consumption. A theoretical model of low-carbon consumption has been developed, and data were collected through a questionnaire survey involving 600 young individuals in the Xizang Autonomous Region. The hypothesized relationships were tested using structural equation modeling techniques.

**Results:**

The findings indicate that the social environment has a significant positive impact on both low-carbon cognition and behavior. Furthermore, low-carbon cognition is shown to positively influence low-carbon behavior. In terms of the relationship between the social environment and low-carbon behavior, it is found that low-carbon cognition acts as a mediating variable. Additionally, it was observed that lower levels of conformity consumption negatively moderate both the relationship between the social environment and low-carbon behavior as well as that between low-carbon cognition and behavior.

**Discussion:**

These findings suggest that engaging young individuals not only fosters environmental awareness but also promotes sustainable consumption, thereby establishing a solid foundation for the protection and enhancement ofour ecological environment. Furthermore, it is essential to disseminate the concept of low-carbon consumerism through various media channels and methodologies. This approach aims to enhance young individuals’ understanding of low-carbon principles, guiding them toward more scientifically informed consumption habits while reducing tendencies for blind conformity.

## Introduction

Global warming has not only led to significant alterations in the natural environment but has also affected people’s production and lifestyles, resulting in shifts in social structures and prompting systemic changes within economic and social domains. Greenhouse gas emissions, in particular, represent a substantial threat to the global climate and are primarily driven by human activities, whether directly or indirectly (Wang et al., 2024). Global warming is fundamentally reshaping our world. It is now incumbent upon various national governments to prioritize and investigate the issue of climate change, as addressing this challenge is essential for advancing global environmental governance.

Promoting the adoption and maintenance of low-carbon consumption behaviors (LCBs) is a critical component of global climate action (Cheng et al., 2023). The foundation for reducing carbon emissions on the consumption side lies in the low-carbon consumption potential of urban residents. Therefore, it is essential to assess and enhance this potential from a maturity perspective—specifically, by evaluating residents’ capabilities and intentions to act responsibly (Wei et al., 2020). Research typically categorizes subjects as urban residents in studies focusing on residents’ LCBs (Ding and Li, 2019). However, existing studies are often insufficient. Much of the research examining factors influencing urban residents’ low-carbon consumption behavior relies on classical theoretical frameworks such as planned behavior theory, value-belief-norm theory, and ABC theory. Nonetheless, findings tend to vary significantly across different time periods, contexts, and sample populations. Consequently, previous conclusions are challenging to generalize or apply widely within academic literature (Ding et al., 2018).

In practice, China’s steadfast commitment to presumptive low-carbon consumption policies provides a robust foundation for further research in this domain. In September 2020, China announced its “double carbon” goals to the international community, making a solemn pledge to peak CO2 emissions by 2030 and achieve carbon neutrality by 2060 (Zeng et al., 2022). Particularly in the Qinghai-Xizang region—often referred to as the “Third Pole of the Earth” and the “Water Tower of Asia”—it is imperative for residents to adopt a low-carbon lifestyle in order to protect the ecological environment. The Chinese government places significant emphasis on ecological and environmental development within Qinghai-Xizang. For instance, Chinese leaders have visited Xizang numerous times, proposing initiatives to ensure ecological safety while actively exploring models and methods for sustainable economic growth. From this perspective, achieving the “double carbon” target in Tibet presents considerable potential for reducing emissions associated with consumption. Individual low-carbon behaviors are essential for decreasing carbon dioxide emissions and enhancing both environmental quality and ecological health. The advancement of a low-carbon circular economy alongside the adoption of green and low-carbon lifestyles is crucial for attaining high-quality economic development on the Qinghai-Xizang Plateau. As an integral component of a low-carbon economy, consumption patterns play a pivotal role in fostering sustainable development within this region (Zhao et al., 2020; Fan and Fang, 2020).

However, due to the combined effects of global changes and human activities (Fan et al., 2015; Li et al., 2018), the Qinghai-Tibet Plateau is facing challenges such as a decline in ecosystem stability and increasing pressure on resources and the environment (Liu et al., 2021; Fayiah et al., 2020). The rise in temperatures and recurrent episodes of extreme weather events on the Qinghai-Tibet Plateau have underscored the urgent need for alterations in economic development strategies and lifestyle choices to protect the region’s ecological integrity (Sun et al., 2016). Consequently, it is essential to address several key questions regarding low-carbon behaviors (LCBs) within the Qinghai-Tibet region: (1) What factors influence low-carbon consumption behavior in this area? (2) Does the social environment in Qinghai-Xizang facilitate the promotion of low-carbon consumption? (3) Can enhancing awareness of low-carbon consumption lead to improved consumption patterns among residents? These inquiries warrant thorough investigation. Furthermore, as a primary consumer demographic, youth play a pivotal role in shaping concepts and behaviors related to low-carbon consumption. Their influence extends significantly to their peers, parents, and other societal groups (Lin and Jia, 2023; Singh et al., 2020). It is crucial to underscore the role of youth in fostering a social culture that advocates for a green and low-carbon society, while also steering the entire community towards adopting and pursuing sustainable lifestyles (Turner, 1991). In our study, the youth demographic primarily comprises college students who are engaged in undergraduate, associate, and graduate programs at institutions of higher education. Typically aged between 18 and 29, this stage marks a significant transition from adolescence to adulthood. As future pillars of societal development, these young individuals make considerable contributions to societal progress through their involvement in social practices, volunteer services, and various initiatives. By participating in these activities, they leverage their academic knowledge to address social challenges and promote harmonious social development. Therefore, this serves as the primary rationale for our focus on this specific group.

Our study develops a model in which low-carbon consumption serves as the dependent variable, the social environment functions as the independent variable, low-carbon cognition acts as the mediating variable, and conformity consumption is treated as the moderating variable. We have opted to employ and innovate upon the theoretical framework of social impact to more effectively analyze the underlying causes of LCBs in the Qinghai-Tibet region. This paper aims to investigate the influencing factors and driving mechanisms behind low-carbon consumption among young individuals on the Qinghai-Tibet Plateau. The objective is to provide insights that will aid in promoting ecological civilization within this region, advocate for widespread adoption of low-carbon consumption practices among residents, and inform policy formulation aimed at achieving carbon neutrality targets on the Qinghai-Tibet Plateau. In accordance with our research design, we will first summarize existing literature; secondly, we will briefly outline our research methods; next, we will present our research findings; finally, we will discuss these results and draw conclusions from our study.

## Literature review and research hypothesis

### The impact of social environment on low-carbon cognition and behavior

Based on social learning theory, social interaction serves as a crucial predictor of low-carbon consumption. The impact of socio-environmental factors on college students’ behavior is significant and far-reaching (Axsen and Kurani, 2012; Carter, 2017). In this study, we define “social environment” variables as the collective external social factors that influence individual decisions and actions regarding low-carbon consumption. These factors primarily include concepts related to low carbon practices, relevant technologies, policies, and products. As government attention towards the greenhouse effect and environmental protection intensifies, public understanding of climate change has deepened. This shift has led to an increasing number of young individuals becoming aware of how their personal consumption affects the environment. To foster a more environmentally friendly lifestyle among youth, governments have cultivated a supportive social atmosphere by implementing policy measures for renewable energy and low-carbon products—such as offering tax incentives and establishing environmental rewards (Song et al., 2022).

The emergence of the sharing economy, driven by advancements in science and technology—such as shared bicycles and vehicles—provides convenient low-carbon travel alternatives that align with the consumption patterns of younger generations. This trend significantly impacts their lifestyle choices (Javaid et al., 2020). Moreover, young individuals are highly engaged with social media and are often influenced by peers, family members, friends, and influencers within their social networks (Mi et al., 2019). Conversations about low-carbon consumption within these networks and the broader societal context can positively shape young people’s attitudes toward sustainable practices. Advocacy for low-carbon consumption from peers, idols, and opinion leaders in their circles is likely to capture their attention while enhancing their sense of identity and increasing their willingness to adopt LCBs (Gerbner, 1972; Wang et al., 2021).

Therefore, the influence of the social environment on youth consumption represents a critical factor. When the social environment consistently advocates for low-carbon consumption and fosters a culture of sustainability, young individuals are more likely to adopt this mindset and align their behaviors with societal norms. This may lead them to engage in conspicuous consumption as a means of expressing their individuality. Conversely, when there is minimal emphasis on low-carbon consumption within society, young people tend to be less inclined to embrace environmentally friendly attitudes and behaviors in their consumer choices. It is evident that the cognitive and behavioral patterns of young individuals regarding low-carbon consumption are significantly shaped by the prevailing social atmosphere. Consequently, this study posits that the social environment exerts a substantial positive influence on both the cognitive processes and behaviors related to low-carbon consumption among young individuals.

Building upon these observations, we propose Hypotheses 1 and 2:

*H1*: The greater the alignment of the social environment towards a cohesive low-carbon atmosphere, the more significantly it can influence the low-carbon cognition of the younger generation.

*H2*: The greater the alignment of the social environment towards a consistent low-carbon atmosphere, the more significantly it can affect the low-carbon consumption behavior of young individuals.

### The effect of low-carbon cognition in LCBs

Cognition refers to the process of acquiring knowledge and understanding events (Barsalou and Lawrence, 2008). An increasing awareness of issues such as carbon emissions and climate change can lead individuals to become more conscious of the environmental impact of their actions. This heightened awareness further stimulates a sense of social responsibility among individuals, prompting them to adopt low-carbon behaviors. Such behaviors may include optimizing travel methods, reducing plastic consumption, favoring environmentally friendly products and services, and avoiding excessive consumption. Research conducted by Anderson indicates that individuals with greater environmental knowledge significantly influence their environmental awareness and behavior (Anderson et al., 1974). Moreover, social psychology posits that human behavior is shaped by various factors including individual emotions, morality, values, and social norms; among these factors, values play a crucial role in guiding individual conduct (Kashima et al., 2021). According to Ajzen (1991) “theory of planned behavior”, an individual’s beliefs, attitudes, subjective norms, and perceived control all contribute to the formation of their behaviors. The higher residents’ awareness regarding the importance of a low-carbon lifestyle—reflecting appropriate values—the greater their engagement in low-carbon behaviors (Yang et al., 2020).

Low-carbon cognition can significantly influence individual behavior; however, the social environment also plays a pivotal role. The information, values, and norms present within the social context can affect an individual’s acceptance of low-carbon cognition and their propensity to translate it into actionable behavior. By fostering and reinforcing a supportive social environment, individuals are more likely to convert low-carbon cognition into tangible actions. According to Gyberg, a low-carbon social atmosphere—characterized by public education and awareness regarding low-carbon practices—is strongly associated with residents’ adoption of low-carbon energy consumption behaviors (Gyberg and Palm, 2009). By guiding individuals through established social norms and moral emotions to cultivate appropriate values while implementing suitable intervention measures, it is feasible for individuals to consistently alter their previous behaviors (Song et al., 2022). Educational initiatives and public awareness campaigns within the social environment can enhance both the dissemination and understanding of low-carbon cognition while aiding individuals in developing sound environmental protection concepts and practices. Through educational institutions, media outlets, and community organizations embedded in the social fabric, there exists significant potential for effectively promoting the transition from low-carbon cognition to sustainable behavioral change.

Based on this analysis, we propose the following hypothesis:

*H3*: The low-carbon cognition among the youth demographic positively influences their low-carbon behaviors.

*H4*: The low-carbon cognition of the youth demographic serves as a mediating factor in the relationship between social and environmental influences and their low-carbon consumption practices.

### The moderating effect of conformity consumption

The consumption patterns of young individuals exhibit significant instability and are highly susceptible to the influences of peer groups and social environments (Wang et al., 2021). Under group pressure, individuals often conform voluntarily to the majority opinion, demonstrating a propensity to align their cognition, perception, and judgment with prevailing public sentiments or behaviors (Banerjee, 1992). They tend to express themselves more freely, embrace new ideas with greater openness, follow trends closely, identify strongly with their peer groups, and display heightened imitation and learning in their behaviors (Hussain et al., 2022; Ali et al., 2021). Conformity can yield both positive and negative outcomes depending on the context in which it occurs (Yu and Sun, 2013). In the domain of consumer behavior, conformity is defined as consumers’ inclination to modify their evaluations, purchase intentions, or actual purchasing behaviors under the influence of similar actions exhibited by others (Kang et al., 2019). Research has shown that teenagers’ novelty-seeking tendencies, the demonstrative effects of celebrities, and consumers’ inclinations to follow trends significantly impact green product consumption (Liu, 2022). Consequently, when low-carbon beliefs or values within a social group converge towards uniformity, varying degrees of conformity may diminish the effect of such consistent beliefs on individual behavior. Studies have indicated that herd mentality negatively moderates the relationship between environmental concern and purchasing behavior (Jingmin and Dong, 2008).

Based on this analysis, we propose the hypothesis 5 and 6:

*H5*: Conformity in consumption negatively moderates the relationship between the social environment and LCBs, with this effect being mediated by low-carbon cognition.

*H6*: Conformity in consumption exerts a negative moderating influence on the direct relationship between social-environmental factors and LCBs.

**Figure 1:**
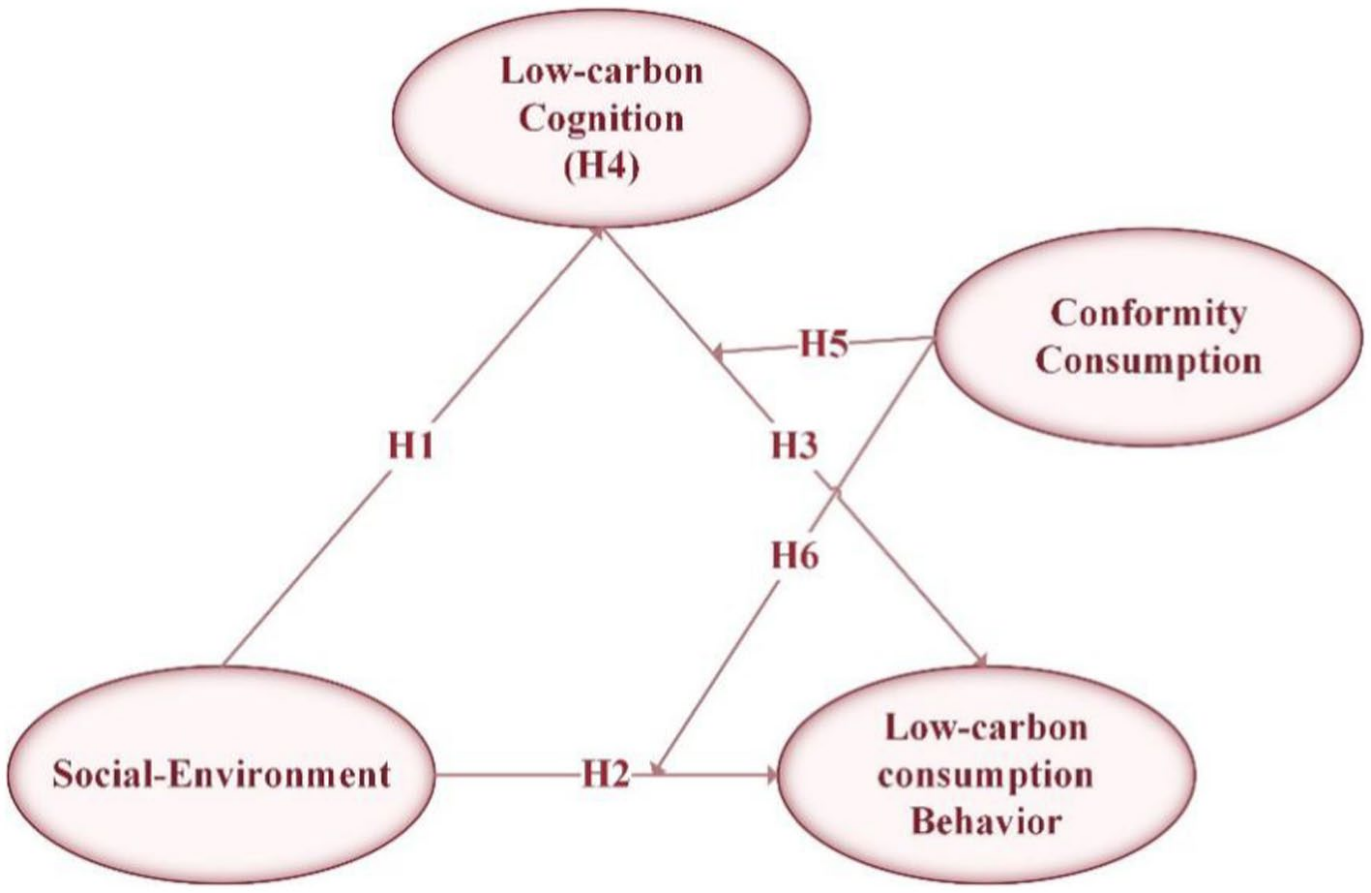
Theoretical analysis framework.

From the analysis presented above, it is evident that the LCBs of young individuals are influenced by a variety of factors. Based on the logical hypotheses underpinning our research, we have developed a theoretical analytical framework for this study, as illustrated in Figure 1. Within this framework, we delineate the impact relationships among social environment, low-carbon cognition, and LCBs. Additionally, we consider conformity consumption as a moderating variable to investigate its potential contribution to the formation of LCBs.

### Data and method

#### Questionnaire design and measurement

All measurement scales employed in this study were derived from established, validated instruments and were adapted to align with specific cultural contexts (Stern, 2000; Dunlap and Riley, 2008) (See Table 1). The items utilized in this research were evaluated using a 5-point Likert scale, ranging from 1 (strongly disagree) to 5 (strongly agree), unless otherwise specified.

**Table 1:** Measurement scales and references.

Latent variable	Indicator	Example item	Reference
Low-carbon cognition (three dimensions, 22 items)	Environmental Awareness (EA)	In my opinion, global warming is primarily caused by excessive carbon dioxide emissions	Bamberg et al. (2003), Chen et al. (2017)
	Low-Carbon Consumption Knowledge (LCCN)		
	Social Responsibility (SR)		
Low-carbon consumption behavior (three dimensions, 20 items)	Low-carbon lifestyle (LCL)	When I travel, I prefer to take the bus or walk rather than take a taxi	Lee et al. (2012)
	Purchasing behavior (PB)		
	Recycling and reusing (RAR)		
Conformity consumption (single dimension, 7 items)	D1-D7	To ensure I purchase the correct brand; I often observe what others are buying and using	Kang et al. (2019), Bearden et al. (1989)
Social environment (single dimension,7 items)	I1-I7	I may consider purchasing low - carbon products if the government provides substantial subsidies for them	Axsen and Kurani (2012), Song et al. (2022)

### Data collection

This study utilized a cross-sectional design, concentrating on young college students in the Xizang Autonomous Region of the Qinghai-Xizang Plateau. In April 2022, a total of 100 surveys were distributed for preliminary investigation, with 95 successfully retrieved. The reliability and validity of the survey instrument were confirmed through testing. During the formal investigation, 600 questionnaires were distributed and recovered, yielding a total of 597 responses. The original data was pre-processed using SPSS version 20.0 software. After excluding 35 invalid samples, we retained 562 valid samples, resulting in an effective response rate of 94%. The analysis of these valid samples (refer to Table 3) reveals that the gender and ethnic proportions among respondents are well-balanced. Additionally, the household registration locations of participants are widely distributed across agricultural and pastoral areas, reflecting the diverse population distribution densities in Xizang due to historical factors and natural environmental conditions. This indicates that our sample is representative and effectively captures overall characteristics within this demographic group.

**Table 2:** Validation and reliability testing.

Latent variable	Indicator	Standardized factor loading	CR	AVE	Cronbach’s α
Low-carbon cognition	EA	0.636	0.898	0.524	0.924
	LCCN	0.809	0.882	0.517	
	SR	0.787	0.883	0.520	
Low-carbon Consumption behavior	LCL	0.583	0.911	0.508	0.899
	PB	0.526	0.891	0.505	
	RAR	0.547	0.866	0.519	
Conformity consumption	D1	0.662	0.880	0.512	0.881
	D2	0.698			
	D3	0.719			
	D4	0.690			
	D5	0.719			
	D6	0.702			
	D7	0.808			
Social- environment	I1	0759	0.982	0.519	0.765
	I2	0.628			
	I3	0.640			
	I4	0.757			
	I5	0.792			
	I6	0.647			
	I7	01796			

EA, Environmental Awareness; LCCN, Low-Carbon Consumption Knowledge; SR, Social Responsibility; LCL, Low-Carbon Lifestyle; PB, Purchasing Behavior; RAR, Recycling and Reusing.

**Table 3:** Descriptive statistics.

Variables	Category	Frequency (N)	Percent (%)
Gender	Male	230	40.9
	Female	332	59.1
Grade	Freshman	143	25.4
	Sophomore	176	31.3
	Junior	153	27.2
	Senior	90	16
Ethnicity	Han	176	31.3
	Zang	376	66.9
	Other	10	1.8
Home location	City	72	12.8
	Town	147	26.2
	Agricultural and pastoral area	343	61
Household registration location	Urban	99	17.6
	Rural	463	82.4
Source of living expenses	Support from parents and family	490	87.2
	Work and study on your own	34	6
	Tutoring or vacation job	23	4.1
	School grants or scholarships	15	2.7

## Result

To gain a more comprehensive understanding of the interconnected relationships among the various constructs, the proposed theoretical model (Figure 1) was analyzed using structural equation modeling. Initially, an assessment of the measurement model encompassing all latent constructs and indicators was performed, followed by an evaluation of the hypothesized structural model.

### Validity, reliability, and fit testing

The Cronbach’s *α* values for each latent variable exceed 0.765, with specific values of 0.924, 0.899, and 0.881 for the respective variables, indicating satisfactory internal consistency (α > 0.765). Reliability and validity results are shown in Table 2.

The load values for low-carbon consumption behavior are 0.583, 0.526, and 0.547, reflecting high consistency among the three factors in the scale. For low-carbon cognition, the load values are 0.636, 0.809, and 0.787, demonstrating strong component consistency as well. The factor loadings between each latent variable and its measurement index range from 0.52 to 0.80; both combined reliability (CR) exceeds 0.6 and average variance extracted (AVE) surpasses 0.5 (see Table 2). These findings indicate robust explanatory power within this questionnaire’s dimensions and affirm its validity. We assessed overall model fit with favorable results: CMIN/df = 2.399 < 3; RMSEA = 0.048 < 0.08; GFI = 0.927 > 0.900; TLI = 0.91 > 0.900; CFI = 0.923 > 0.900—most indices suggest strong explanatory power.

Using Harman’s single-factor exploratory analysis via SPSS software to assess common method bias revealed that the first factor explains only 15% of variance without rotation—well below the significant threshold of 40%. This indicates a low level of common methodological bias. The root mean square of AVE values for all latent variables exceeds their correlation coefficients with other variables, suggesting robust discriminant validity among selected latent variables.

### LCBs under the influence of the social environment

A simple mediation model was employed to investigate the mediating effect of low-carbon cognition on the relationship between social environment and LCBs while controlling for gender and grade (see Tables 4, 5). The results indicate that the social environment significantly predicts LCBs. Furthermore, even when accounting for the mediating role of low-carbon cognition, this predictive effect remains significant. Additionally, the social environment exerts a substantial influence on low-carbon cognition, which in turn significantly predicts LCBs. Moreover, both the direct effect of social environment on LCBs and the mediating effect of low-carbon cognition yield a bootstrap confidence interval at 95% that does not include zero. This finding suggests that not only can the social environment directly predict LCBs but it can also do so indirectly through its influence on low-carbon cognition.

**Table 4:** Results of the hypothesis test.

Path	B	β	SE	*t*	*p*
Social-environment → low-carbon cognition	0.511	0.599	0.062	8.2	***
Low-carbon cognition → low-carbon consumption behavior	0.157	0.247	0.055	2.849	0.004
Social-environment → low-carbon consumption behavior	0.189	0.349	0.051	3.702	***
Low-carbon cognition x conformity consumption → low-carbon consumption behavior	−0.117	−0.218	0.034	−3.413	***
Conformity consumption → low-carbon consumption behavior	−0.055	−0.117	0.028	−2.007	0.045
Social-environment x conformity consumption → low-carbon consumption behavior	−0.084	−0.151	0.034	−2.454	0.014

***p < 0.01, **p < 0.05, *p < 0.1.

**Table 5:** Test results of the moderating effect.

Path	Conformity levels	Effect	Bootstrapping 95%CI	
			Lower	Upper
Mediation path				
Social-environment → low-carbon cognition → low-carbon consumption behavior	Low	0.279	0.164	0.420
	High	0.018	−0.133	0.165
Direct path				
Social-environment → low-carbon consumption behavior	Low	0.500	0.304	0.720
	High	0.197	−0.152	0.503

Following this, we adjusted the latter half of the mediation model and the direct path. The mediated model was analyzed while controlling for gender and grade. The findings indicate that when incorporating the regulatory variable of conformity consumption into the model, both the interaction term between social environment and conformity consumption, as well as the interaction term between low-carbon cognition and conformity consumption, exhibit significant predictive effects on low-carbon behaviors (LCBs). Furthermore, the standardized path coefficient is negative, indicating a substantial adverse impact. This suggests that conformity consumption not only negatively moderates the direct influence of social environment on low-carbon consumption behavior but also adversely affects the role of low-carbon cognition in predicting low-carbon consumption behavior through mediating variables.

**Figure 2:**
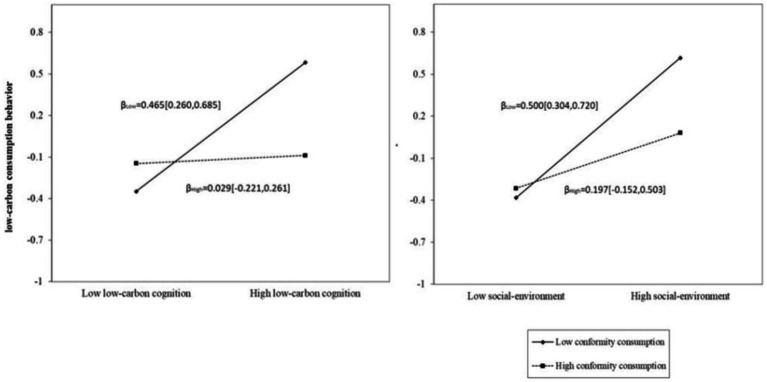
The moderating effect of varying levels of conformity on the mediated influence pathway of low-carbon cognition and on the direct influence of LCBs.

Further combined with a simple slope analysis (see Figure 2), it was found that the impact of the social environment on low-carbon consumption behavior, as well as the influence of the social environment mediated by low-carbon cognition on low-carbon consumption behavior, is contingent upon levels of conformity. A high level of conformity in consumption exhibits a weaker moderating effect (95% CI: −0.152 to 0.503, −0.133 to 0.165). In contrast, a low level of conformity in consumption demonstrates a significant negative moderating effect (95% CI: 0.304 to 0.720, 0.164 to 0.420). The results indicate that lower levels of conformity in young people’s consumption are associated with diminished influences from the social environment on their LCBs; similarly, reduced levels of conformity also correlate with decreased impacts from the social environment mediated by low-carbon cognition on their LCBs.

This finding aligns with previous studies (Lim et al., 2023). Conformity refers to the tendency of individuals to be influenced by group behaviors, perspectives, or values in order to adhere to group norms or expectations. Low levels of conformity indicate that individuals are less susceptible to the influence of societal groups and are more likely to engage in independent thought and action. For those exhibiting low conformity, the impact of the social environment on their low-carbon consumption behavior is either weak or virtually non-existent. Similarly, among young people with low levels of conformity, there is a diminished effect of the social environment mediated by low-carbon cognition when predicting their low-carbon consumption behavior. Conversely, no significant moderating effect is observed for young individuals displaying high levels of conformity.

## Discussion and conclusion

Based on data collected from 600 young individuals in the Qinghai-Xizang Plateau, this study examines and theoretically analyzes the relationship between low-carbon behaviors (LCBs) and three latent variables: social-environmental factors associated with low-carbon consumption, low-carbon cognition, and conformity behavior within youth groups. Six research hypotheses are proposed. Through hypothesis testing utilizing structural equation modeling analysis, the results indicate that the model is valid and supports all six research hypotheses. The analysis reveals that: (1) The social environment positively influences both the low-carbon cognition and LCBs of young people; (2) Low-carbon cognition has a positive effect on the LCBs of young individuals; (3) Low-carbon cognition serves as a mediating variable in the relationship between social environment and LCBs among youth. Furthermore, it is found that (4) at lower levels of conformity among young people, there exists not only a negative moderating effect on the direct influence of social environment on low-carbon consumption behavior but also a detrimental moderation of the intermediary variable—low-carbon cognition—on LCBs. For youth group LCBs, both social environment and low-carbon cognition emerge as critical variables. It is essential to recognize that individual levels of conformity exert varying effects on these two variables’ influences and should not be overlooked.

The youth population plays a pivotal role as both participants and leaders in the future of social development, with their consumption concepts and behavioral habits significantly influencing this trajectory. By engaging in low-carbon consumption practices, young individuals can cultivate environmental awareness and sustainable consumption principles, thereby establishing a solid foundation for the protection and enhancement of the ecological environment in the future. Simultaneously, as consumers, the choices made by young people will shape market demand. By opting for environmentally friendly and low-carbon products and services, they can incentivize businesses to expedite their transition towards green production methods. This shift not only promotes carbon reduction but also fosters ecological preservation on the production side (Liu et al., 2019). The Qinghai-Tibet Plateau serves as a crucial ecological barrier within China; thus, safeguarding its ecological environment is imperative. Consequently, actively promoting a low-carbon economy while encouraging young people to participate in low-carbon consumption practices will positively contribute to achieving sustainable development goals. In light of these insights, this article proposes several strategies and recommendations aimed at enhancing Low-Carbon Behaviors (LCBs) among youth in the Qinghai-Tibet Plateau region: specifically targeting other social groups to foster widespread adoption of LCBs that align with achieving “dual carbon” objectives.

Firstly, the social environment exerts a direct and positive influence on the low-carbon behaviors (LCBs) of young individuals. As members of society, young people continuously adjust their actions to align with their social surroundings, which includes aspects such as conformity and socialization. Peer pressure within this environment also significantly affects individual behavior (Mi et al., 2019). When the social atmosphere collectively fosters a consistent culture of low-carbon consumption, it profoundly encourages young people’s behavioral choices in this regard. Therefore, enhancing the LCBs among youth should commence with an emphasis on improving the social environment; this represents a crucial entry point for addressing this issue. The social environment serves as a pivotal factor influencing young individuals and primarily manifests through its role in guiding and exemplifying rational low-carbon consumption practices among college students via a cohesive societal atmosphere. An increase in factors that advocate for and promote low-carbon consumption within the community will provide more exemplary models and accurate guidance regarding consumption concepts for young people (Yin and Shi, 2021). Even if individuals lack comprehensive knowledge about low-carbon practices, they are likely to follow trends toward sustainable consumption when society uniformly endorses such behaviors. Conversely, if societal attitudes towards low-carbon consumption are negative or if there is an absence of robust external support for these initiatives, it can adversely affect young people’s engagement with sustainable practices. Thus, advocating for low-carbon principles and promoting environmentally friendly consumerism emerge as critical variables shaped by societal contexts. In advancing a low-carbon economy and encouraging eco-friendly products within the Qinghai-Xizang Plateau region, it is imperative to widely disseminate the concept of low-carbon consumerism through diverse media channels and methods while fostering participation from all segments of society to cultivate a unified atmosphere that supports LCBs.

Secondly, low-carbon cognition can directly predict the low-carbon consumption behaviors of young people and simultaneously serves as a mediating factor in the relationship between social-environmental influences and the low-carbon behaviors (LCBs) of this demographic. Consequently, low-carbon cognition plays a pivotal role in shaping LCBs among youth. By enhancing their understanding of low-carbon principles, young individuals can grasp the impact of carbon emissions on the environment, recognize how their consumption choices relate to these emissions, and learn strategies for reducing carbon footprints through altered consumption habits and selecting green products. This awareness fosters a deeper appreciation for the significance of adopting a low-carbon lifestyle while bolstering environmental consciousness and willingness to take action (Xia et al., 2018). Moreover, cultivating low-carbon awareness is essential for transforming LCBs. It motivates young people to actively opt for low-carbon products and services during their purchasing activities, thereby contributing positively to environmental protection efforts and carbon reduction initiatives (Hu et al., 2021). Additionally, low-carbon cognition shapes youths’ attitudes toward sustainable consumption practices as well as their sense of social responsibility; they perceive such actions as vital avenues for engaging in environmental stewardship and promoting sustainable development. A positive orientation towards low-carbon principles not only enhances young people’s knowledge about sustainability but also cultivates an affirmative ecological value system that guides them in transitioning from traditional high-carbon consumption patterns to more sustainable lower-carbon lifestyles.

Finally, this study found that the behavior of conforming to others directly influences low-carbon behaviors (LCBs) within the social environment. Furthermore, the social environment characterized by low-carbon cognition serves as a mediator and exerts a negative moderating effect on LCBs. Further analysis revealed that when conformity levels among young individuals are low, there is a detrimental moderating effect on the relationship between these two factors. The trait of conformity can exhibit both positive and negative dimensions, contingent upon the context and its implications. In certain circumstances, conformity may facilitate individuals in learning and adapting to new environments, information, or cultures by allowing them to acquire valuable experiences and knowledge from others. This trait increases an individual’s likelihood of becoming an influencer in social change by leading trends and group behaviors, which can yield positive societal impacts. However, excessive conformity may result in blind imitation and herd mentality, ultimately impairing independent thinking abilities and judgment while rendering individuals susceptible to misinformation or harmful behaviors. A study examining motivations for healthy food choices indicated that demand driven by health consciousness—rational decision-making based on an objective understanding of dietary health—is relatively stable (Ding and Li, 2019). Whereas the consumption of organic food, influenced by conformity psychology, often exhibits fluctuations in response to changing consumer trends, resulting in greater instability (Wang et al., 2023). Among youth groups, there are more pronounced manifestations of comparison-based consumption phenomena that may lead to irrational behaviors such as blind conformity in consumption patterns devoid of self-awareness. For instance, the pursuit of novelty or uniqueness during consumption processes—without regard for practical value—could adversely affect more rational and scientifically informed low-carbon behaviors (LCBs). Therefore, when guiding young individuals on the Qinghai-Tibet Plateau towards adopting LCBs, it is imperative for governments to enhance the promotion of low-carbon knowledge while effectively raising awareness about environmental issues within this demographic group.

Continuously enhancing their understanding of low-carbon concepts will guide individuals toward scientific rationality in their consumption habits while mitigating blind conformist behaviors. Elevated levels of low-carbon awareness can empower individuals to make more rational and independent sustainable consumption decisions. Low-carbon awareness plays a pivotal role in promoting sustainable consumption practices, irrespective of the influence exerted by societal factors.

If young people acquire accurate knowledge regarding what constitutes “low-carbon” and comprehend the interdependence between human production activities and environmental climate resources, they will be motivated to adopt pro-low carbon consumption inclinations. To engage effectively in low-carbon consuming practices, it is essential to understand what defines “low-carbon” and which types of consumption qualify as “low-carbon.” Additionally, recognizing the interrelationship between human activities and environmental climate resources is crucial. Only then can individuals diminish non-independent preferences such as blind adherence or ostentation, thereby fostering a collective commitment to being rational, persistent practitioners and advocates for sustainable low-carbon consumption practices.

However, it is essential to acknowledge the limitations of this study. The research primarily concentrates on young individuals and does not address other demographic groups. We recognize that college students constitute only a subset of this broader demographic. While college students may exhibit distinct characteristics regarding social influences, cognitive development, and consumption patterns, they also represent a significant segment of the youth population—particularly in studies examining behavioral change and consumption trends. We believe that their inclusion offers valuable insights into the wider youth demographic; however, we recommend that future research explore additional subgroups within the youth population, such as non-college youths or young professionals, to further assess the generalizability of our findings. Lastly, the analysis of LCBs in this study predominantly focuses on individual consciousness while neglecting the influence of public policies. In conclusion, future research will continue to emphasize these areas.

## Data Availability

The raw data supporting the conclusions of this article will be made available by the authors, without undue reservation.
